# Serological profile of infectious bovine rhinotracheitis, bovine viral diarrhea, leptospirosis, toxoplasmosis, and neosporosis in beef cattle herds in Tocantins, Northern Brazil

**DOI:** 10.1007/s11259-025-10989-1

**Published:** 2025-11-25

**Authors:** José C. Ribeiro-Júnior, Bruna Alexandrino, Juliana T. T. Fritzen, Beatriz S. L. Nino, Katyane S. Almeida, Juliane Ribeiro, João L. Garcia, Lucienne G. P. Giordano, Alice F. Alfieri, Luciana B. S. B. da Costa, Amauri A. Alfieri

**Affiliations:** 1 Department of Preventive Veterinary Medicine, College of Veterinary Medicine, The Ohio State University (OSU), 1900 Coffey Road, Columbus, OH 43210 USA; 2 Microbiology Laboratory, School of Veterinary Medicine, Universidade Federal do Norte do Tocantins (UFNT), BR-153, KM 112, Araguaína, Tocantins 77804-970 Brazil; 3 Laboratory of Animal Virology, Department of Preventive Veterinary Medicine, Universidade Estadual de Londrina (UEL), Londrina, Paraná Brazil; 4 Protozoology Laboratory, Department of Preventive Veterinary Medicine, Universidade Estadual de Londrina (UEL), Londrina, Paraná Brazil; 5 Leptospirosis Laboratory, Department of Preventive Veterinary Medicine, Universidade Estadual de Londrina (UEL), Londrina, Paraná Brazil

**Keywords:** Bovine diseases, Serology, Bovine alphaherpesvirus 1. bovine viral diarrhea virus, *Leptospira* spp., Neospora caninum

## Abstract

Monitoring the health of cattle herds is essential for establishing prophylactic measures and reducing their impact on the production chain, especially in regions where beef cattle form the economic base. This study aimed to determine the serological profile of infectious bovine rhinotracheitis (IBR), bovine viral diarrhea (BVD), leptospirosis, toxoplasmosis, and neosporosis in beef cattle slaughtered in a federally inspected slaughterhouse in the state of Tocantins, northern Brazil. A total of 614 serum samples from 70 herds were evaluated for the presence of antibodies to Bovine alphaherpesvirus-1 and Bovine viral diarrhea virus by virus neutralization test, 12 serovars of *Leptospira* spp. by microscopic agglutination test, and to *Toxoplasma gondii* and *Neospora caninum* by indirect immunofluorescence reaction. The rates of seropositive animals for IBR, BVD, leptospirosis, toxoplasmosis, and neosporosis were 90.4, 50.8, 63.2, 6.2, and 7.3%, respectively. For herds, 100% had at least one animal serum positive for IBR and leptospirosis. Considering the seropositive rates for the five infectious diseases observed in slaughtered cattle, the number of beef cattle in Tocantins, and the intrinsic conditions of the etiological agents being by regional seasonality, it is essential to implement or improve animal health surveillance, prevention, and biosecurity programs to ensure better productivity and economic sustainability of beef cattle herds in the state.

## Introduction

Infectious Bovine Rhinotracheitis (IBR), Bovine Viral Diarrhea (BVD), and Leptospirosis are significant infectious diseases associated with reproductive disorders in both beef and dairy cattle worldwide (Moore et al. 2021; İnce and Ayaz 2023). *Neospora caninum* infections, which cause reproductive problems, are more common in dairy cattle (Barros et al. 2021) but have also been reported in beef herds (Santos et al. 2010). Although very sporadically, toxoplasmosis has also been linked to reproductive failures in cattle (Gomes et al. 2020).

Given that IBR, BVD, and Leptospirosis are considered endemic in Brazilian beef cattle herds, and that Neosporosis and Toxoplasmosis are rarely evaluated, determining the serological profile of herds is essential. It helps in understanding the epidemiology of these infections, assessing the relative importance of each disease in terms of the frequency of seropositive animals and herds, identifying risk factors for infection, and defining control, prophylactic, and biosecurity measures to prevent the introduction and intra-herd spread of pathogens.

Although these infections have a significant impact on reproductive efficiency in beef cattle, conducting serological assessments during the breeding season and fixed-time artificial insemination adds stress to the animals due to the handling required for blood collection. This can negatively affect animal welfare and, consequently, cow productivity (Chenais and Fischer 2018).

Over time, BoAHV-1, BVDV, and *Leptospira* spp. have evolved strategies to persist in herds. BoAHV-1, an alphaherpesvirus, establishes latency by maintaining its genome in the nuclei of neurons. When latency ends, the virus may be shed in secretions and excretions, potentially infecting seronegative animals and triggering clinical symptoms. BVDV perpetuates mainly through the birth of immunotolerant, persistently infected (PI) calves, resulting from intrauterine infection with the non-cytopathic strain between 60 and 120 days of gestation. These PI animals - representing 1 to 3% of births - shed the virus throughout their lives in all secretions and excretions (Ostler and Jones 2023). *Leptospira* spp. causes chronic kidney infections and is shed intermittently in the urine, contaminating water, food, and facilities, thereby posing a risk to susceptible animals (Lilenbaum and Martins 2014).

Therefore, in beef cattle herds, performing serological tests on pasture-finished steers at slaughter offers a useful alternative for determining the circulation of BoAHV-1, BVDV, *Leptospira* spp., *N. caninum*, and *Toxoplasma gondii* within herds. This study was designed to evaluate the serological profile of these reproductive pathogens in slaughtered cattle and herds in the state of Tocantins, northern Brazil, to support disease control, prevention, and biosecurity programs.

## Materials and methods

### Experimental design

For significant representativeness, the minimum sampling outlined was 385 serum samples, using a confidence level of 95%, a margin of error of 5%, an estimated prevalence of 50% (for greater experimental scope), and an estimated cattle herd population of the state of 11.3 million animals (IBGE 2017) in software EpiInfo™ v.7.2 (https://www.cdc.gov/epiinfo).

### Sampling

A total of 614 bovine zebu serum samples were obtained from 70 beef cattle herds in the State of Tocantins, distributed among 18.7% (26/139) of the total municipalities, mainly from the north of State. From each herd, a minimum of eight animals were sampled according to the batch sent for slaughter. Seven herds were slaughtered at different times, and for this reason, ≈ 16 animals were evaluated from these herds.

Blood samples were collected between October and November 2019 from a cattle slaughterhouse in the municipality of Araguaína, under a federal inspection regime, during bleeding and after the animal was stunned by the percussive penetrative method. The sampling for origin, age, and sex was conducted randomly. All serum samples from females were older than 24 months. In the antemortem documentation, the animals evaluated did not have a history of IBR, BVD, and leptospirosis vaccination. However, the absence of this information in the slaughterhouse does not mean that the animals did not receive these vaccines, since the information for these diseases is not mandatory for slaughterhouses in Brazil.

Approximately 10 mL of whole blood was collected from each animal in a conical polypropylene tube. After natural coagulation, the tubes were sent to the Microbiology Laboratory of Universidade Federal do Norte do Tocantins (UFNT), where they were centrifuged at 3,500 x *g* for 7 min, and 1 mL aliquots of serum were stored at − 20 °C.

### Serological analysis

All serum samples were sent in isothermal boxes to the Animal Virology, Leptospirosis, and Veterinary Protozoology laboratories of the Universidade Estadual de Londrina (UEL), Londrina, Paraná, for serological analyses.

The virus neutralization (VN) test was performed to detect the presence of neutralizing antibodies against Bovine alphaherpesvirus-1 (BoAHV-1) and Bovine viral diarrhea virus (BVDV), as outlined in the Manual of Diagnostic Tests and Vaccines for Terrestrial Animals of the World Organization for Animal Health (WOAH 2024).

The microscopic agglutination test (MAT) for *Leptospira* spp. was performed according to Faine (1999) against the serogroups Australis (serovar Bratislava), Autumnalis (serovar Butembo), Ballum (serovar Castellonis), Canicola (serovar Canicola), Grippothyphosa (serovar Grippothyphosa), Icterohaemorrhagiae (serovars Icterohaemorrhagiae and Copenhageni), Pomona (serovar Pomona), Pyrogenes (serovar Pyrogenes), Sejroe (serovar Hardjo and Wolffi), and Tarassovi (serovar Tarassovi).

The presence of antibodies anti-*Neospora caninum* and anti-*Toxoplasma gondii* was evaluated by indirect immunofluorescence reaction (RIFI), according to Conrad et al. (1993) and Camargo (1973), respectively.

### Statistical analysis

All analyses were performed using positive and negative controls for each replicate. Data were analyzed using the SAS 9.0 statistical software (SAS 2020).

### Ethics in the use of animals

This study was authorized by the Ethics Committee on the Use of Animals (CEUA) at the Federal University of Tocantins (Process no. 23.101.003/2019-52).

## Results and discussion

Table 1 presents the serological profile of each infectious disease in cattle and herds.

**Table 1:** Frequency of seropositive animals and herds for of infectious bovine rhinotracheitis (IBR), bovine viral diarrhea (BVD), toxoplasmosis, neosporosis and leptospirosis in the Tocantins State, Brazil

	Total (*n* = 614)			Herds (*n* = 70)		
	No.	Frequency (%)	CI (95%)^1^	No.	Frequency (%)	CI (95%)^1^
IBR	555	90.39	88.6–92.72	70	100	100–100
BVD	312	50.81	46.86–54.77	63	90	82.97–97.03
Toxoplasmosis	38	6.19	4.28–8.09	28	40	28.52–51.48
Neosporosis	45	7.33	5.27–9.39	29	41.43	29.89–52.97
Leptospirosis	388	63.19	54.89–71.49	70	100	100–100

^1^ CI: Confidence interval

### IBR

In this study, 100% of cattle herds and 90.39% of the animals were IBR-seropositive. Considering the latency characteristics of BoAHV-1 infection, health programs could be implemented (Alfieri et al. 2019). Due to the high risk of embryonic and fetal mortality, the reproduction management of the farms should be re-evaluated. Fernandes et al. (2019) related the reproductive losses, sharing equipment and facilities with other farms, and herd size > 23 animals as the main influential risk factors for the occurrence of IBR in beef herds in Northeastern Brazil. In the South, Dias et al. (2013) identified that beef herds, natural mating, animal purchase, pasture leasing, the existence of calving pens, and documented abortion cases in the last 12 months were the main risk factors for IBR.

This relative diversity of risk factors and regional differences in the peculiarity of cutting between regions justify the differences in the incidence of IBR in Brazil, even within the same states. Studies carried out with beef cattle during different periods in Paraná, southern Brazil, found that the frequency of IBR can oscillate from 50.8% (Medici et al. 2000) to 71.3% (Dias et al. 2013). In Paraíba, in Northeastern Brazil, the prevalence of IBR was reported at 84% for herds and 73% for beef animals (Fernandes et al. 2019). As in the present study in the Legal Amazon region, in the state of Pará, Costa et al. (2012) reported a prevalence of 33.5% for IBR in 1,920 Nellore cattle from two herds.

Of the animals sampled, 90.4% (555/614) tested positive for IBR. High antibody titers (≥ 64) were recorded in 46.3% (257/555) of these animals, while 34.5% (192/555) had medium titers (16 to 32). Animals with low (2 to 8) or non-reactive titers had similar percentages (≈ 9%). These findings indicate that the virus is present in the region.

### BVD

For BVD, 90% of herds were positive (Table 1) and from the 312 (50.81%) serum samples positives, 42.63% (133) show intermediary (40 to 80) titers, and 30.77% (96) and 26.6% (83) were positive with high (≥ 160) and low (10 to 20) titers, respectively. Costa et al. (2012) reported 40% of positive cows in two properties in the state of Pará, near Tocantins. In Colombia, a region with seasonal conditions similar to the region of this study, González-Bautista et al. (2021) reported 42.5% of BVD prevalence and related breed, age (> 4 years), management practices, and pasture lease as risk factors associated with BVD.

BVD is one of the main infectious diseases associated with reproductive problems in cattle (Alfieri et al. 2019). Also in Tocantins, Chicoski et al. (2023) used a limited number of samples (75 serum samples) and observed 72% of reactive animals. These same authors observed a frequency of 51.8% (656/1,266) of BVD for the total serum samples of beef cattle sampled in several Brazilian States (Mato Grosso, Mato Grosso do Sul, Goiás, São Paulo, and Minas Gerais) in addition to Tocantins.

Detection of BVD-seropositive animals or variations in antibody titers in herds that have no vaccination history is a risk factor and indicates economic losses, with the herd estimated to incur €58 in losses per animal per year (Gunn et al. 2004). Thus, it can be considered that the productivity and performance of beef cattle herds in the state are negatively influenced by the high seroprevalence of BVDV infections. The adoption of health programs such as good production practices, including vaccination against the main diseases that affect cattle within the breeding system, promotes animal health and increases cattle production (Alfieri et al. 2019).

### Neosporosis

In total, 41.43% of the herds and 7.33% of the animals evaluated were positive for neosporosis, which is lower than the reported 13.69% of positive animals reported in a meta-analysis from China (Ying et al. 2022). Spontaneous abortion, history of previous pregnancies, age older than one year, and extensive management are risk factors for bovine neosporosis (Ying et al. 2022). These risk factors are consistent with the conditions of the animals evaluated in this study in the state of Tocantins. Cows destined for slaughter, as in this study, may have related reproductive problems, of which neosporosis may also be associated. *Neospora* infection-positive cows are 2.42 times more likely to abort than infection-negative cows (Ying et al. 2022). No pre-slaughter information regarding the occurrence of abortions was identified in the cows evaluated in the present study.

### Toxoplasmosis

Toxoplasmosis had serological positivity testing in 6.19% of the total evaluated population and 40% of the herds (Table 1). In addition to the low prevalence in animals, the immune response against *T. gondii* is not directly related to the presence of viable cysts in the animal’s carcass, limiting the risk of transmission of the etiological agent and disease through meat consumption (Opsteegh et al. 2019). A study by Blaga et al. (2019) also reported that beef consumption is not related to positivity for toxoplasmosis in humans, but showed that older age can influence the higher seroprevalence of *T. gondii*.

Interestingly, in the present study, the serological profile of neosporosis and toxoplasmosis was low for animals but intermediate for herds (Table 1), demonstrating the wide dissemination of the etiological agents in different municipalities in Tocantins. In the state of Amazonas, also Northern Brazil and the Amazon Region, however, serological positivity of 30.9% of 1,073 animals and 93.6% of 47 cattle herds for toxoplasmosis has been reported, and an epidemiological investigation found that the number of animals on the farm and the presence of domestic cats are risk factors (Azevedo Filho et al. 2020).

### Leptospirosis

**Fig. 1:**
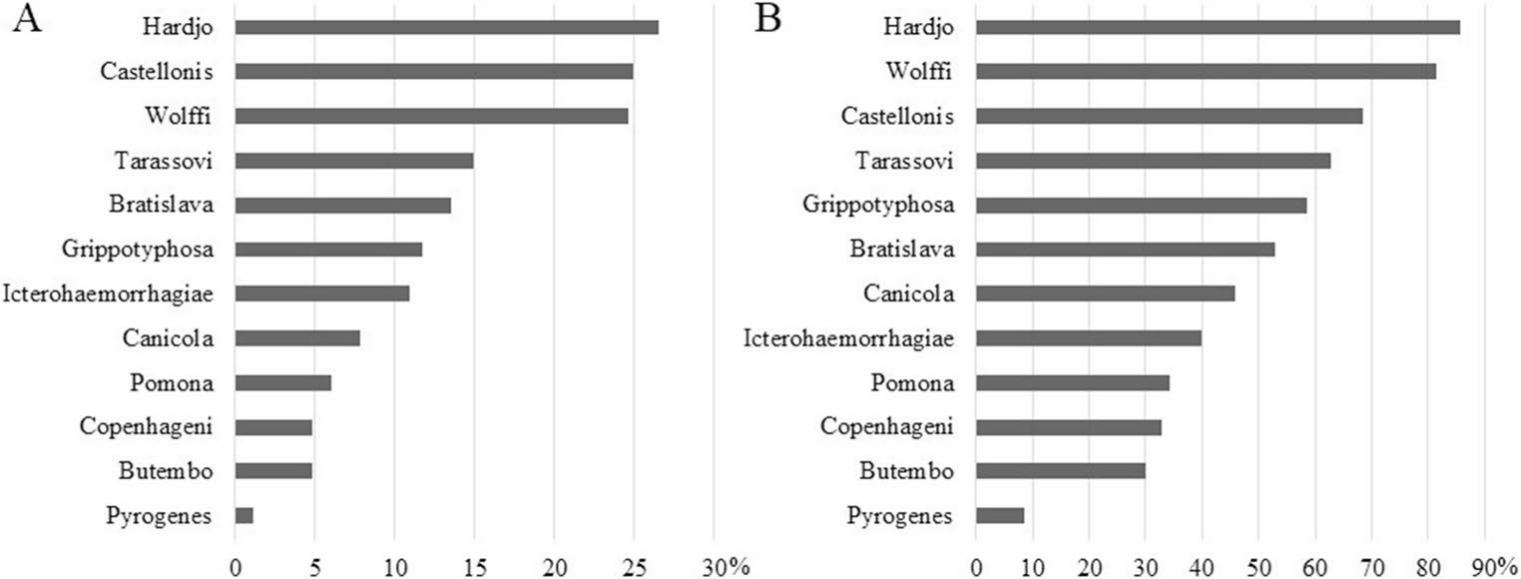
Rates (%) of seropositive beef cattle animals (**A**, *n* = 614) and herds (**B**, *n* = 70) for 12 serovars of *Leptospira* spp. in Tocantins State, Brazil

Figure 1 presents the results of the frequency of each *Leptospira* serovar for animals (1 A) and herds (1B). Of the total of 614 animals evaluated, 388 (63.19%) presented a positive reaction with some titer for at least one of the 12 serovars studied. For the herds, 100% (70/70) presented at least one animal reagent to some serovar.

In Tocantins, the cattle vaccines marketed include the serovars Canicola, Grippotyphosa, Hardjo, Icterohaemorrhagiae, and Pomona (Leptoferm^®^, CattleMaster^®^ 4 + L5 and CattleMaster^®^ Gold FP 5/L5, Zoetis Brazil). Since it was not possible to retrieve the vaccination history, it is not possible to state whether the titers observed for these serovars result from prophylactic programs. However, among the 614 samples, all that presented some titer for the serovars present in the vaccines also presented titers for serovars not included. Only 2 (0.32%) samples presented titers simultaneously for the five serovars present in the vaccines; however, they also presented titers for Wolffi, Copenhageni, Bratislava, and Butembo. Therefore, it is very likely that the samples that comprised this study were from animals not vaccinated for leptospirosis or, if they were vaccinated, the titers were not detectable.

A great diversity of *Leptospira* serovars circulating in cattle from Tocantins was observed. All 12 serovars of the 10 serogroups studied were identified in cattle and herds with greater or lesser prevalence (Fig. 1). The least incident serovar was Pyrogenes (1.14%) in 7 (10%) herds, and the most incident was Hardjo (26.55%) in the 60 (85.7%) herds evaluated (Fig. 1B).

## Conclusion

A high prevalence of IBR, BVD, and leptospirosis has been observed in beef cattle and herds from Tocantins. This main finding may or may not be related to vaccination programs, the history of which could not be accessed by the present study. Considering the importance of the beef cattle production chain for this State, as well as the negative impacts on reproductive parameters of heifers and cows, in particular, those caused by IBR, BVD, and leptospirosis, control and prophylaxis measures of microbial infections must be adopted and monitored. Reproductive health is essential for good production rates and, mainly, the productivity rates of the beef cattle chain in the State of Tocantins. To reach this goal, risk factors for infections must be mitigated, and well as must be implemented vaccination programs for mainly BoAHV-1, BVDV, and *Leptospira* infection control.

## Data Availability

Data is unavailable due to ethical restrictions.
